# Informetric Analysis of Highly Cited Papers in Environmental Sciences Based on Essential Science Indicators

**DOI:** 10.3390/ijerph17113781

**Published:** 2020-05-26

**Authors:** Qian Ma, Yandan Li, Yan Zhang

**Affiliations:** 1 Tianjin University Library, Tianjin University, Tianjin 300350, China; 2 Information Science Institute, Tianjin University, Tianjin 300350, China; 3 School of Environment, Tsinghua University, Beijing 100084, China; liyandan@mail.tsinghua.edu.cn; 4 State Key Laboratory of Biogeology and Environmental Geology, School of Water Resources and Environment, China University of Geosciences-Beijing, Beijing 100083, China; yanzhang@cugb.edu.cn

**Keywords:** environmental sciences, informetric analysis, highly cited paper, Essential Science Indicators

## Abstract

Highly cited papers in the Essential Science Indicators database refer to papers with citations in the top 1% of all papers in a research field, and they are considered to be symbols of scientific excellence and top performance of the past ten years. This study provided an informetric analysis of 7791 highly cited papers in the environmental sciences category during 2009–2019. Informetric indicators and visualization tools were applied to evaluate and present the performances of journals, countries/territories, institutions, top cited papers, and research hotspots. The results showed that the cumulative number of publications has increased exponentially, suggesting strong development of the environmental sciences category. There were 211 journals publishing highly cited papers, with Energy & Environmental Science as the leading journal. The USA ranked first with the highest number of publications and occupied the core position in the collaboration network, while Mainland China took the first place in independent research output. Review articles have an obvious advantage in terms of achieving high citations. “Adsorption”, “climate change”, and “heavy metal” were the most frequent keywords, with “microplastic” rising rapidly as a new research frontier in recent years. Five research hotspots were visualized from highly cited papers via cluster analysis.

## 1. Introduction

Environmental science is an emerging interdisciplinary field relating social needs and environmental problems. It involves many aspects, including air, soil, water, and ecology [1,2]. With the rapid development of the economy and industrialization process, our environment has suffered great pressure from population growth and the enhancement of production activities [3,4]. As a result, various environmental problems are increasingly prominent, such as the greenhouse effect, acid rain, ozone layer destruction, groundwater pollution, and ocean acidification [5,6]. In order to solve these environmental problems, great efforts (e.g., manpower, material, and financial resources) have been invested by governments and all sectors of society [7]. In recent years, environmental science has experienced remarkable development, marking a new stage of human beings’ understanding, utilization, and modification of the environment [8].

Essential Science Indicators (ESI) is an analytical database based on Clarivate Analytics’ Web of Science Core Collection (WoS). Publication counts and citation data for ESI are derived from journals indexed in the Science Citation Index-Expanded (SCIE) and the Social Sciences Citation Index (SSCI) of WoS during the past ten years [9]. In ESI, papers are divided into 22 research fields, and an article is assigned to only one field. ESI database reveals the performance and development as well as leading institutions, countries/territories, journals, and papers in a research field. For a specific ESI research field, highly cited papers (HCPs) refer to these papers with the citations entered the top 1% of all papers in each year. HCPs are considered to be a symbol of scientific excellence and top performance.

Informetric analysis of HCPs can help to identify significant research trends and the most influential research papers, while avoiding the bias of publishing year on citations. Such analysis has been performed in many fields, including economics and business [10], geosciences [11], and operations research and management science [12]. In the field of environmental sciences, Khan and Ho (2012) analyzed 88 selected articles with more than 500 citations, ignoring the publication year of those papers [13]. The citation count of a paper is always influenced by its publication year. Generally speaking, the earlier a paper is published, the more citations it will have. This paper aimed to provide a systematic informetric analysis of HCPs in the environmental sciences category. The objectives of this study are as follows: (1) evaluate the research performance of research outputs and the top cited paper in each year; (2) assess the top players in terms of journal, country/territory, and institution; (3) mapping hotspots and research developments with keywords analysis and cluster analysis.

## 2. Methodology

### 2.1. Data Source

The publication data in this study were obtained from the Clarivate Analytics’ ESI and WoS database. According to 2018 Journal Citation Reports (JCR), environmental sciences is one of their 254 Web of Science Categories. The retrieval formula (WC = “Environmental Sciences” and PY = “2009–2019”) was used to search the publications in the SCIE and SSCI database (here, “WC” refers to Web of Science Category, “PY” refers to year published). The document types were restricted to “Article” and “Review”. Retrieval results showed that there were 559,498 SCIE/SSCI papers in the environmental sciences category. Among these, 7791 papers were marked as HCPs. In correspondence to HCPs, those 559,498 SCIE/SSCI papers were labelled as “all papers” (APs) in this study. Here, it should be noted that the ESI database is updated every two months, and the latest update time for this study was in January 09, 2020. Thus, the data source is relatively steady. The data of the 7791 HCPs were downloaded and analyzed comprehensively.

### 2.2. Data Analysis

Informetric analysis is a powerful and important tool in evaluating scientific performance and development of a research field [14,15,16,17,18]. Many indicators were applied to assess the scientific performance, including publication indicators (such as the number of publications, publication share), citation indicators (such as total citations, citations per paper, H-index, percentage of papers receiving at least one citation), and journal indicators (such as the impact factor, JIF Quartile, Immediacy Index). Analysis of keywords has been widely used to identify hotspots and research trends in recent years [19,20,21]. VOSviewer, a visualization software, was utilized to construct the collaboration network and perform the co-word cluster analysis [22].

**Figure 1:**
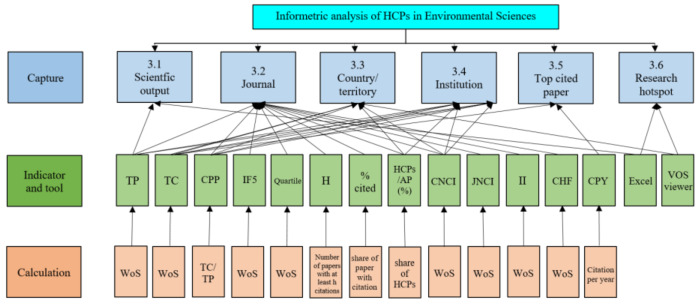
Analysis structure and indicators used in the study. TP: the number of publications; TC: total citations; CPP: citations per paper; IF5: 5-year impact factor; Quartile: JIF Quartile; H: H-index; % Cited: percentage of papers received at least one citation; HCPs/APs (%): percentage share of HCPs; CNCI: Category Normalized Citation Impact; JNCI: Journal Normalized Citation Impact; II: Immediacy Index; CHF: Cited Half-Life; CPY: citations per year.

Figure 1 illustrates the indicators used in this study. Among them, H-index was introduced by Hirsch [23] and can be defined as follows: if the H-index of a journal is h, then it has h papers that are cited at least h times and its other publications’ citations are not more than h. HCPs/APs (%) means the percentage share of HCPs, and its world average value is 1%. This percentage reflects the overall standard of papers of one item (e.g., research category, journal, country/territory, institution). The Category Normalized Citation Impact (CNCI) is an unbiased indicator of impact irrespective of research category, publication year, and document type. The CNCI of a document is calculated by dividing the actual citations by the expected citation rate for documents with the same document type, year of publication, and research category. A CNCI value of one represents performance at par with the world average of one research category. If CNCI > 1, the citation performance is considered above the world average; and if CNCI < 1, it is considered below average. Similar to CNCI, the Journal Normalized Citation Impact (JNCI) is defined as citation impact normalized for publication year, document type, and journal in which the document is published. If JNCI > 1, the citation performance is considered above the world average of its publishing journal, and if JNCI < 1, it is considered below average. The Immediacy Index (II) is the average number of times a paper is cited in the year it is published. Cited Half-Life (CHF) is an indicator of the quality of a scientific journal and is defined as the median age of the papers that were cited in the JCR year. Generally speaking, the higher the CHF, the more influential that journal is thought to be.

## 3. Results and Discussion

### 3.1. Characteristics of Scientific Output

**Figure 2:**
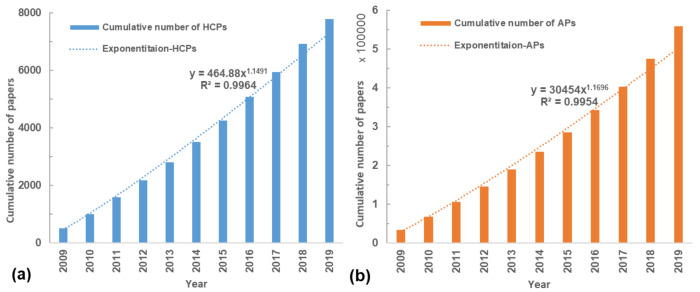
Cumulative number of (a) highly cited papers (HCPs) and (b) all papers (APs) in the environmental sciences category.

Over the past ten years, the output of APs increased significantly from 33,396 in 2009 to 84,078 in 2019, with an average annual growth rate of 9.81%. Simultaneously, the number of HCPs increased from 512 in 2009 to 872 in 2019, with an average growth rate of 5.95%. The HCPs accounted for 1.04%–1.56% (average of 1.39%) of all papers (i.e., APs) from 2009 to 2019 in the environmental sciences category, which is obviously larger than the world average share of HCPs (1%). Figure 2 shows the cumulative number of HCPs and APs from 2009. One can see that the cumulative number of HCPs and APs fit Price’s curve with R^2^ = 0.99, suggesting the exponential growth of cumulative amount of HCPs and APs. These results indicated the strong development of research output and the high impact potential of the environmental sciences category.

### 3.2. Distribuiton of Journals

There were 410 academic journals publishing papers in the environmental sciences category during 2009–2019, among which 211 journals published HCPs. Table 1 lists the top ten productive journals with the highest number of HCPs and APs. Five journals, including Journal of Hazardous Materials (JHM), Science of the Total Environment (STE), Environmental Science & Technology (EST), Journal of Cleaner Production (JCP), and Atmospheric Chemistry and Physics (ACP) belonged to both the top ten productive journals of HCPs and APs.

As shown in Table 1, Energy & Environmental Science (EES) had the highest quantity of published HCPs (837), accounting for 10.74% of total HCPs. The following four journals were JHM (718, 9.22%), STE (494, 6.34%), EST (461, 5.92%), and JCP (453, 5.81%). For citations per paper (CPP), EES, EST, NCC, and ACP had the best performance with more than 200 citations.

STE was the most productive journal in the environmental sciences category; it published 22,848 APs, followed by Sustainability (17,219), EST (16,805), JCP (16,206), and ESPR (16,097). EES had the largest CPP of 115, followed by NCC (76).

When comparing the citation indicators of HCPs with those of APs, one can see that the CPP, CNCI, and JNCI of HCPs were all larger than those of APs for the above-mentioned 15 journals, verifying the high quality of HCPs. The sixth column was HCPs/APs (%), which reflects the overall standard of papers published in a journal. Among the 15 leading journals listed in Table 1, EES and NCC had the highest percentages of 23.76% and 21.4% respectively, which were significantly larger than the others 13 journals. As for impact factor, EES and NCC had the largest IF5 among all journals categorized in the environmental sciences category. EES had the highest H-index (298), followed by EST (215). Twelve of the 15 leading journals had an H-index larger than 100. These results indicated the high research quality of leading journals in the environmental sciences category as a whole.

The three journals with HCPs/APs (%) < 1% (i.e., Sustainability, ESPR, and IJERPH) had relatively low numbers of HCPs, % cited, immediacy index, IF5, H-index, and cited half-life when compared to the others 12 leading journals in Table 1. Moreover, the CNCI of APs that published in three journals were less than 1, implying that their average paper quality was below the average level of the environmental sciences category. On the other hand, the large JNCI of HCPs indicated the large difference of paper quality between HCPs and APs. Those results suggested that improvement of paper quality is still needed for the three journals. It should be noted that the cited half-life and impact factor of IJERPH showed a steady increase, from 2.3 and 1.605 in 2001 to 3.6 and 2.468 in 2018, respectively, which indicated a gradual improvement of paper quality in recent years to some extent.

### 3.3. Research Performance by Country/Territory

The 7791 HCPs in the field of environmental sciences were published from 138 countries/territories around the world. Table 2 shows the top 15 countries/territories with the highest number of HCPs. The sum of these 15 countries/territories’ HCPs was 7057, including 90.58% of all HCPs. Of these 15 countries/territories, eight were in Europe, four in Asia, two in North America, and one in Oceania. The USA ranked as the most productive country/territory with an obvious advantage (2812, 36.09%). The second most productive country/territory was Mainland China (2322, 29.80%). The remaining top 15 productive countries/territories were England (1122, 14.40%), Germany (844, 10.83%), Australia (800, 10.27%), Canada (667, 8.56%), the Netherlands (640, 8.21%), France (551, 7.07%), Spain (494, 6.34%), Italy (463, 5.94%), Switzerland (450, 5.78%), Sweden (351, 4.51%), India (304, 3.9%), South Korea (281, 3.61%), and Japan (262, 3.36%). Thus, the USA and Mainland China had dominant positions in the scientific outputs of HCPs. Moreover, the USA and Mainland China had the highest academic influence with total citations of 557,240 and 302,799, respectively. Results indicated that the two countries/territories contributed not only nearly 60% of all HCPs, but also most of the HCPs’ total citations. For the percentage HCPs/APs (%), Switzerland had the highest value of 3.83%, followed by the Netherlands (3.78%) and England (3.14%). The USA and Mainland China had similar HCPs/APs (%) of ~2%. India had the poorest performance on HCPs/APs (%), CPP, and CNCI among these 15 countries/territories, indicating that its academic influence could be further strengthened by improving paper quality and visibility [24].

**Figure 3:**
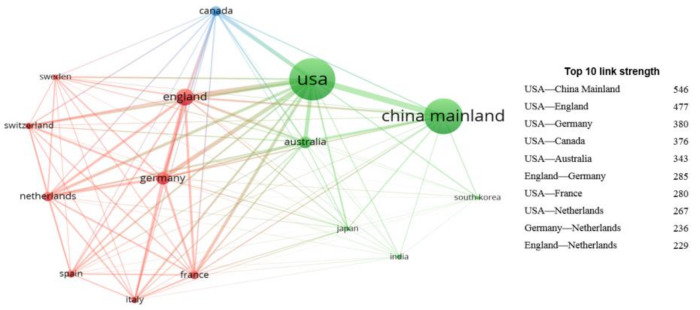
Collaboration network of top 15 countries/territories in the environmental sciences category.

The international collaborations of these 15 countries/territories were studied. As shown in Table 2, the USA was the major collaborative partner of the others 14 countries/territories. The USA also took a leading position in the output of internationally collaborative HCPs (1727), accounting for 61.42% of all HCPs in the country. Mainland China took the first place in terms of output of independent research with 1203 HCPs, and the USA took the second place with 1085 independent HCPs. International collaboration contributed more than 60% of all HCPs for each country/territory, except Mainland China (48.19%). On one hand, it suggested the high openness of these 14 countries/territories in the environmental sciences category. On the other hand, it means that the HCPs of these 14 countries/territories depended highly on other countries/territories, indicating their relatively weak independent research ability. Figure 3 illustrates the collaboration network of these top 15 countries/territories, where the size of node represents the number of HCPs and the thickness of line represents link strength. As shown in Figure 3, the collaboration network was strongly connected, with all countries/territories collaborated with each other. In the network, the biggest node was the USA with 2812 HCPs. The highest link strength was between the USA and Mainland China with 546 collaborative HCPs. There were noticeable collaborative subnetworks too, including “USA–England–Germany”, “USA–England–Australia”, “USA–England–Netherlands”, “USA–England–France”, “USA–Germany–Netherlands”, and “USA–Germany–France”. Mainland China, as the second largest node, mainly collaborated with the USA and had relatively little collaborations with the others 13 countries/territories. Among the 10 strongest links, the USA occurred seven times. These results indicate that the USA occupies a core position in the collaboration network.

### 3.4. Research Performance of Institutions

At the institution level, there were 6174 institutions publishing HCPs in the environmental sciences category. Table 3 presents the top 15 productive institutions associated with country/territory, TP, HCPs/APs (%), TC, CPP, and CNCI. Among them, six institutions were in the USA, three in Mainland China, and one each in France, Germany, the Netherlands, Australia, Switzerland, and Spain. The Chinese Academy of Sciences ranked first with 629 HCPs, accounting for 8.07% of all 7791 HCPs in the environmental sciences category. The remaining institutions with more than 200 HCPs were the University of California with 409 HCPs (5.25%), followed by CNRS (284, 3.65%), the Helmholtz Association (250, 3.21%), and the United States Department of Energy (236, 3.03%). Among the top 15 institutions, the National Aeronautics & Space Administration (NASA) had the highest HCPs/APs (%) of 4.91%. The University of Chinese Academy of Sciences had the poorest performance not only in HCPs/APs (%), but also in CPP and CNCI, indicating that its academic influence should be further developed.

### 3.5. Top Cited Paper in Each Year

Considering that there is citation bias between old and new papers, the top cited paper in each year was studied. Table 4 displays the top cited paper, including title, total number of citations, document type, publication year, citation per year, and published journal, in each year.

The paper in the environmental sciences category titled “A comparative assessment of decision-support tools for ecosystem services quantification and valuation” had the highest number of citations, with 10,518. Second, the paper titled “Preferred Reporting Items for Systematic Reviews and Meta-Analyses: The PRISMA Statement” was cited 4458 times. Of the top 11 papers during 2009–2019, six were articles and five were review papers. Review papers accounted for 45.45% of the top cited papers during 2009–2019; i.e., article papers accounted for 54.55% of the top cited papers. Whereas, in all 7791 HCPs, the percentage of review papers was reduced to 24.11%, and that of article papers was increased to 75.89%. In all 559,498 APs in the environmental sciences category, the percentage of review papers was just 4.14%, and that of article papers was 95.86%. These results indicated that review papers in the environmental sciences category have an obvious advantage in achieving high citations. Those 11 papers were published in six different journals, among which six papers were from EES, and one each from ES, JCE, WR, RSE, and BC. Thus, EES also had distinct advantage in publishing the top cited paper in each year.

### 3.6. Research Hotspots of HCPs in Environmental Sciences

**Figure 4:**
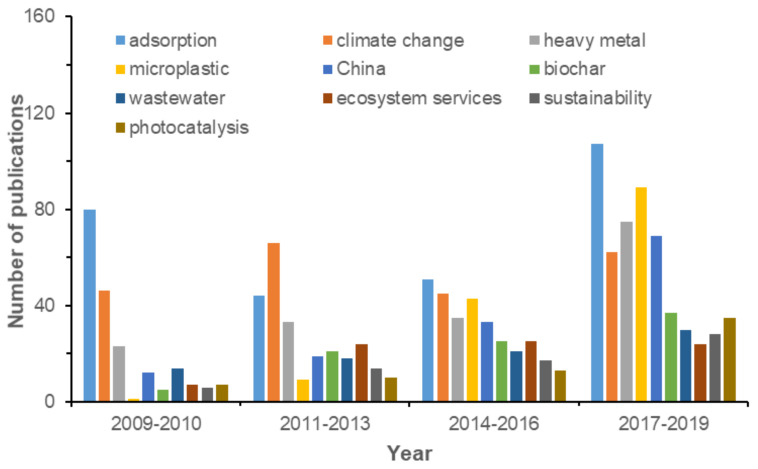
Top 10 high-frequency keywords of HCPs in the environmental sciences category.

Author keywords are the essence and core of each piece of literature and reveal the areas of most interest to researchers. By analyzing author keywords, one can identify research emphasis and development trends [36,37]. As shown in Figure 4, “adsorption”, “climate change”, “heavy metal”, “microplastic”, and “China” were the leading author keywords with no less than 100 occurrences during 2009–2019. Adsorption is a key process to study the environmental fate of contaminants and an important and effective method for the removal of environmental pollutants. Climate change has great influences on the global environment, inducing many environmental problems such as global warming, acid deposition, and ozone depletion. In 2013, carbon dioxide, the leading culprit in climate warming, exceeded 400 ppm for the first time. It sounded the alarm for climate warming again, and the corresponding number of HCPs reached a peak in 2013 with 28 HCPs. “China” was the top frequently used country as an author keyword in the field of environmental sciences. It should be noted that only one article used “microplastic” as an author keyword in 2009, but the number of papers using “microplastic“ as an author keyword rose to second place in the period of 2017–2019. This result indicated that “microplastic” is not only a research hotspot, but also new frontier in the environmental sciences category.

**Figure 5:**
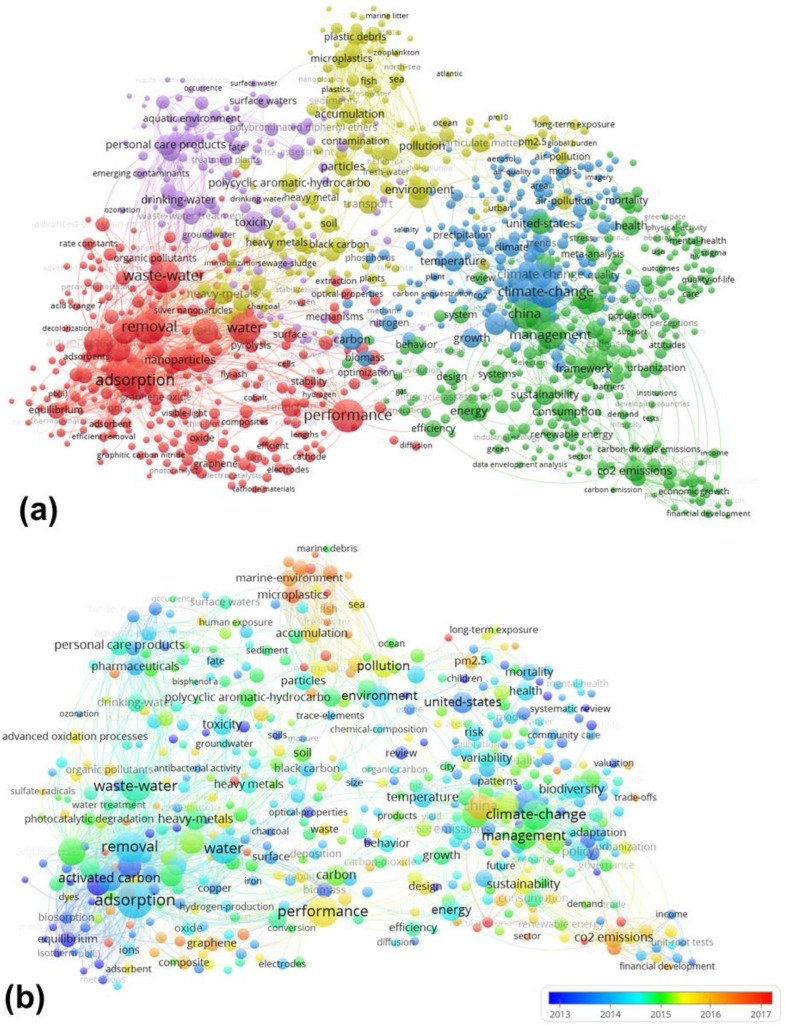
(**a**) Cluster analysis and (**b**) overlay visualization of paper keywords.

Co-word cluster analysis can identify relationships between keywords, which can further reflect the research hotspots of HCPs in the environmental sciences category [19]. The cluster analysis of author keywords and Keywords plus was performed with VOSviewer software, and the results are shown in Figure 5a. According to the cluster analysis result, the keywords were aggregated into five clusters. Cluster 1 (red items in Figure 5a) involved the treatment of environmental pollutions and corresponding kinetic processes, which had the main keywords of “adsorption”, “removal”, “degradation”, “sorption”, “oxidation”, “activated carbon”, “aqueous solution”, “nanoparticle”, and “waste water”. Cluster 2 (green items) focused on urban ecological and environmental pollution management, including keywords such as “CO_2_ emission”, “management”, “sustainability”, “urbanization”, “energy”, “policy”, “conservation”, and “economic growth”. Cluster 3 (blue items) referred to research on climate change, including keywords: “climate change”, “carbon”, “temperature”, “precipitation”, “emission”, “impact”, and “model”. Cluster 4 (yellow items) covered research on toxic pollutants and their impact on the ecological environment, including the main keywords of “heavy metal”, “cadmium”, “particle”, “pm2.5”, “particulate matter”, “microplastic”, “accumulation”, “contamination”, “soil”, “fish”, and “sea”. Cluster 5 (purple items) focused on wastewater treatment with keywords including “personal care product”, “wastewater”, “drinking water”, “aquatic environment”, “toxicity”, “pharmaceutical”, and “tandem mass spectrometry”. Figure 5b is the overlay visualization of author keywords and Keywords plus, and the color of a keyword is determined by the average of publication year. For example, “microplastic”, “marine environment”, “graphene”, and “CO_2_ emission” occurred frequently in recent years, while “kinetics” and “sorption” mainly occurred in earlier years. From Figure 5a, one can find emerging hotspots of HCPs in the environmental sciences category, such as “microplastic” associating with “marine environment”, “CO_2_ emission” with the energy consumption process, and performance of graphene in the environment.

Based on the above keyword analyses, one can see that the treatment of environmental pollutants and corresponding kinetic processes was the biggest hotspot with the highest number of clustered keywords. However, regeneration and reuse of adsorbent and its secondary contamination are still lacking in systematic research. Secondly, as a novel environmental pollutant resistant to degradation, microplastics are widely known to be ideal carriers for organic pollutants and heavy metals, and have widespread distribution in the environment (e.g., marine environment, territorial soil, atmosphere environment, land and sea life, and even drinking water). Nevertheless, there remain some key technical points that need to be solved urgently in microplastic pollution, including its efficient separation and identification, and reliable source identification techniques and models. Moreover, research on microplastics has been mainly conducted in marine environments, and other environments should receive more attention in the future. In the last decade, biochar has played an increasingly important role in environmental remediation, carbon sequestration, and soil modification. How to enhance the adsorption capacity, and stability of biochar is an important but difficult task. In addition, as an important component of global water cycle, groundwater can transport a large number of pollutants into surface waters (river water, lake water, and ocean), which would potentially contribute to eutrophication, acidification, and the occurrence of red tides. Thus, groundwater needs to be further discussed and considered in the study of environment pollution.

## 4. Conclusions

This study adopted informetric methods to analyze HCPs during 2009–2019 in the environmental sciences category based on the ESI database. Insights were made into various aspects, including research output, the most productive journals, countries/territories, institutions, top cited papers, and research hotspots, with systematic informetric analysis and visualization tools. The results showed an exponential growth of the cumulative amount of both HCPs and APs, suggesting strong development of the environmental sciences category. EES had the highest number of HCPs and top cited papers, as well as the highest IF5, H-index, and HCPs/APs (%). Sustainability, ESPR, and IJERPH ranked as the top 10 productive journals in terms of APs in the environmental sciences category, but have lower paper quality. The USA and Mainland China were the leading countries/territories, and they had the highest number of HCPs, APs, and academic citations. The USA had a dominant position in the collaboration network of the top 15 productive countries/territories, and Mainland China took the first place in independent research output. As for institutions, the Chinese Academy of Sciences had the highest number of HCPs and total citations. Analysis of top cited paper in each year suggested that review papers have an obvious advantage in achieving high citations.

“Adsorption”, “climate change”, “heavy metal”, “microplastic”, and “China” were the most frequent author keywords. The study of microplastics is a new research frontier with rapid growth in recent years. Research hotspots of HCPs in the environmental sciences category mainly include the treatment of environmental pollutants and corresponding kinetic processes, urban ecological and environmental pollution management, climate change, toxic pollutants and their impact on the ecological environment, and wastewater treatment. The results of this study can provide insight for scholars in the field of environmental sciences, including comprehensive understanding of leading journals, countries/territories, and institutions in the research field, which can further guide paper submission, collaboration, and academic exchange. From these high-quality papers, one can also learn useful information, such as theory, tools, and methods, and trace the research hotspots and frontiers to find critical issues for scholars and governments.

There are some limitations to this study. First, Clarivate Analytics’ Web of Science Categories were used in data retrieval. Therefore, papers published in journals not categorized in the environmental science category were not included. Furthermore, this study focused on informetric analysis of HCPs, with only a preliminary analysis of APs. In addition, HCPs are just a part of papers published in the environmental sciences category; therefore, the cluster analysis of HCPs cannot be used to identify research loopholes or gaps.

## Figures and Tables

**Table 1:** Leading journals of HCPs and APs in the environmental sciences category.

Journal	Rank		TP		HCPs/APs (%)	CPP		CNCI		JNCI		Quartile	IF5	H	II	CHF	% Cited
	APs	HCPs	APs	HCPs		APs	HCPs	APs	HCPs	APs	HCPs						
EES	35	1	3523	837	23.76	115	295	5.26	12.77	1	2.38	Q1	32.83	298	5.54	4.7	99
JHM	8	2	12,668	718	5.67	35	156	1.65	6.73	1	3.96	Q1	7.34	190	2.30	8	96
STE	1	3	22,848	494	2.16	15	88	1.64	10.22	1	5.74	Q1	5.73	140	1.68	4.4	88
EST	3	4	16,805	461	2.74	35	220	1.66	9.47	1	5.66	Q1	7.87	215	1.14	7.8	95
JCP	4	5	16,206	453	2.8	15	91	1.47	7.16	1	4.92	Q1	7.05	125	1.69	2.9	88
EPc	15	6	7723	350	4.53	24	118	1.70	7.98	1	4.65	Q1	5.46	134	1.12	7.1	93
WR	13	7	7860	254	3.23	34	184	2.15	9.80	1	4.46	Q1	8.42	162	1.66	8.1	94
NCC	118	8	1182	253	21.4	76	203	5.74	14.30	1	2.47	Q1	25.17	150	4.12	4.2	97
RSE	29	9	4111	238	5.79	38	178	2.39	10.83	1	4.47	Q1	8.79	148	2.07	8.8	95
ACP	9	10	8633	213	2.47	28	203	1.62	10.04	1	5.99	Q1	6.20	155	1.23	6	94
Sustainability	2	43	17,219	34	0.2	3	57	0.58	10.84	1	19.03	Q2	2.80	53	0.75	2.2	59
ESPR	5	25	16,097	61	0.38	8	118	0.76	10.09	1	13.05	Q2	3.21	78	0.68	3.2	81
Chemosphere	6	13	14,712	172	1.17	18	125	1.34	8.51	1	5.89	Q1	5.09	129	1.13	6.7	91
IJERPH	7	43	13,931	34	0.24	6	140	0.75	8.79	1	11.33	Q1	2.95	80	0.56	3.6	68
EPt	10	14	8323	166	1.99	20	153	1.50	9.39	1	6.11	Q1	6.15	124	1.32	6.6	88

APs: all papers; HCPs: highly cited papers; TP: the number of publications; CPP: citations per paper; CNCI: Category Normalized Citation Impact; JNCI: Journal Normalized Citation Impact; Quartile: JIF Quartile; IF5: 5-year impact factor; H: H-index; II: Immediacy Index; CHF: Cited Half-Life; % Cited: percentage of papers received at least one citation. EES: Energy & Environmental Science; JHM: Journal of Hazardous Materials; STE: Science of the Total Environment; EST: Environmental Science & Technology; JCP: Journal of Cleaner Production; EPc: Energy Policy; WR: Water Research; NCC: Nature Climate Change; RSE: Remote Sensing of Environment; ACP: Atmospheric Chemistry and Physics; Sustainability: Sustainability; ESPR: Environmental Science and Pollution Research; Chemosphere: Chemosphere; IJERPH: International Journal of Environmental Research and Public Health; EPt: Environmental Pollution.

**Table 2:** Characteristics of the top 15 productive countries/territories.

Country/Territory	TP				HCPs/APs (%)	TC	CPP	CNCI	ICP (%)	MC (P)
	HCPs (R)	%	APs (R)	%						
USA	2812 (1)	36.1	139,409 (1)	24.9	2.02	557,240	198	10.59	1727 (61)	Mainland China (546)
Mainland China	2322 (2)	29.8	116,149 (2)	20.8	2	302,799	130	9.99	1119 (48)	USA (546)
England	1122 (3)	14.4	35,693 (3)	6.4	3.14	201,666	180	10.23	869 (77)	USA (477)
Germany	844 (4)	10.8	32,013 (5)	5.7	2.64	148,892	176	10.61	720 (85)	USA (380)
Australia	800 (5)	10.3	28,763 (6)	5.1	2.78	134,133	168	10.34	672 (84)	USA (343)
Canada	667 (6)	8.6	32,182 (4)	5.8	2.07	128,792	193	10.63	537 (81)	USA (376)
Netherlands	640 (7)	8.2	16,912 (11)	3	3.78	121,118	189	11.25	544 (85)	USA (267)
France	551 (8)	7.1	23,251 (10)	4.2	2.37	99,505	181	9.93	487 (88)	USA (280)
Spain	494 (9)	6.3	27,637 (7)	4.9	1.79	92,385	187	10.16	387 (78)	USA (160)
Italy	463 (10)	5.9	23,348 (9)	4.2	1.98	85,244	184	10.35	393 (85)	USA (188)
Switzerland	450 (11)	5.8	11,744 (18)	2.1	3.83	96,559	215	11.87	372 (83)	USA (217)
Sweden	351 (12)	4.5	12,882 (16)	2.3	2.72	65,002	185	9.58	302 (86)	USA (148)
India	304 (13)	3.9	24,057 (8)	4.3	1.26	45,052	148	8.07	197 (65)	USA (72)
South Korea	281 (14)	3.6	15,516 (13)	2.8	1.81	47,481	169	10.35	192 (68)	USA (84)
Japan	262 (15)	3.4	16,709 (12)	3	1.57	50,748	194	11.62	221 (84)	USA (146)

TP: the number of publications; HCPs: highly cited papers; APs: all papers; R: rank of each country/territory according to the number of publications; %: percentage share of papers in all HCPs or APs; TC: total citations; CPP: citations per paper; CNCI: Category Normalized Citation Impact; ICP (%): the number and percentage of international collaborative HCPs, MC (P): major collaborative country/territory (number of collaborative HCPs).

**Table 3:** Characteristics of the top 15 productive institutions.

Institute	Country/Territory	TP		HCPs/APs (%)	TC	CPP	CNCI
		HCPs (R)	Aps (R)				
Chinese Academy of Sciences	Mainland China	629 (1)	27,513 (1)	2.29	96,318	153	9.91
University of California System	USA	409 (2)	14,620 (2)	2.8	82,546	202	10.34
Centre National de la Recherche Scientifique (CNRS)	France	284 (3)	11,290 (3)	2.52	55,445	195	10.4
Helmholtz Association	Germany	250 (4)	7635 (5)	3.27	43,464	174	10.33
United States Department of Energy	USA	236 (5)	6319 (6)	3.73	56,689	240	11.61
Tsinghua University	Mainland China	184 (6)	4820 (13)	3.82	26,603	145	9.76
Wageningen University & Research	Netherlands	183 (7)	3868 (20)	4.73	35,260	193	11.49
State University System of Florida	USA	166 (8)	6271 (7)	2.65	33,226	200	11.08
Harvard University	USA	163 (9)	4491 (16)	3.63	28,329	174	10.81
Commonwealth Scientific & Industrial Research Organisation (CSIRO)	Australia	159 (10)	3502 (26)	4.54	28,597	180	11.69
National Oceanic Atmospheric Admin (NOAA)- USA	USA	155 (11)	3459 (29)	4.48	27,763	179	10.36
National Aeronautics & Space Administration (NASA)	USA	153 (12)	3117 (38)	4.91	31,266	204	11.53
ETH Zurich	Switzerland	152 (13)	3368 (31)	4.51	29,373	193	10.54
University of Chinese Academy of Sciences, CAS	Mainland China	145 (14)	9206 (4)	1.58	17,375	120	9.16
Consejo Superior de Investigaciones Cientificas (CSIC)	Spain	144 (15)	5713 (8)	2.52	29,548	205	9.75

APs: all papers; HCPs: highly cited papers; TP: the number of publications; TC: total citations; CPP: citations per paper; CNCI: Category Normalized Citation Impact.

**Table 4:** Top cited paper in each year.

Title	TC	Document Type	PY	CPY	Journal
Worldwide decline of the entomofauna: A review of its drivers [25]	84	Review	2019	84	BC
Rational design of electrocatalysts and photo(electro) catalysts for nitrogen reduction to ammonia (NH3) under ambient conditions [26]	212	Article	2018	106	EES
Google Earth Engine: Planetary-scale geospatial analysis for everyone [27]	475	Article	2017	158	RSE
Cesium-containing triple cation perovskite solar cells: improved stability, reproducibility and high efficiency [28]	1727	Article	2016	432	EES
Supercapacitor electrode materials: nanostructures from 0 to 3 dimensions [29]	984	Review	2015	197	EES
Formamidinium lead trihalide: a broadly tunable perovskite for efficient planar heterojunction solar cells [30]	1496	Article	2014	249	EES
A comparative assessment of decision-support tools for ecosystem services quantification and valuation [31]	10,518	Article	2013	1503	ES
Na-ion batteries, recent advances and present challenges to become low cost energy storage systems [32]	1877	Article	2012	235	EES
Challenges in the development of advanced Li-ion batteries: a review [33]	2876	Review	2011	320	EES
Recent developments in photocatalytic water treatment technology: A review [34]	2137	Review	2010	214	WR
Preferred Reporting Items for Systematic Reviews and Meta-Analyses: The PRISMA Statement [35]	4458	Review	2009	405	JCE

TC: total citations; PY: publication year, CPY: citations per year. BC: Biological Conservation EES: Energy & Environmental Science; RSE: Remote Sensing of Environment; ES: Ecosystem Services; WR: Water Research; JCE: Journal of Clinical Epidemiology.
